# Patterns and associated factors of accelerometer-measured physical activity in the metropolitan areas of Singapore and Berlin – comparative analysis of the Singapore population health studies and the German National Cohort (NAKO)

**DOI:** 10.1186/s12889-025-22922-x

**Published:** 2025-05-21

**Authors:** Paul Kittner, Thore Bürgel, Claire Marie Goh Jie Lin, Stefan N. Willich, Thomas Keil, Falk Müller-Riemenscheider, Lilian Krist

**Affiliations:** 1 Center for Digital Health, Berlin Institute of Health (BIH), Charité-Universitätsmedizin Berlin, Berlin, Germany; 2 Saw Swee Hock School of Public Health, National University of Singapore, Singapore, Singapore; 3 Institute of Social Medicine, Epidemiology and Health Economics, Charité-Universitätsmedizin Berlin, Berlin, Germany; 4 Institute of Clinical Epidemiology and Biometry, University of Würzburg, Würzburg, Germany; 5 State Institute of Health I, Bavarian Health and Food Safety Authority, Erlangen, Germany; 6 Yong Loo Lin School of Medicine, National University of Singapore, Singapore, Singapore

**Keywords:** Accelerometry, Physical activity, Singapore, Germany, Cohort study, Urban health

## Abstract

**Background:**

Physical activity (PA) plays a critical role in preventing non-communicable chronic diseases. However, engagement in PA differs widely between countries. The aim of this study was to examine PA patterns and associated factors in the urban populations of Singapore and Berlin, Germany.

**Methods:**

This study used harmonized data from the Singapore Population Health Studies and the study center “Berlin-Mitte” of the German National Cohort Study (NAKO). PA was assessed with hip-worn accelerometers. Raw tri-axial accelerometry data was processed using the GGIR-R-package and classified into moderate-to-vigorous PA (MVPA), light PA (LPA), and inactivity. Multivariable regression analyses were applied to analyze associations between sociodemographic and lifestyle factors and PA intensities.

**Results:**

The analyses included 1,195 (57.2% female, 46.6 ± 13.7 years) participants from Singapore and 2,060 (49.3% female, 49.9 ± 12.6 years) from Berlin. Singaporean participants engaged in more MVPA (+ 14.1 min [10.6;17.5] [95%-confidence interval]) and LPA (+ 66.2 min [57.4;74.9]) and less time inactive (-80.2 min [-90.0;-70.4]) than Berlin participants. In Singapore, more MVPA was associated with Chinese ethnicity and being employed, more LPA with male sex, normal weight, lower education and being married, while more inactivity was associated with female sex, overweight and non-smoking. In Berlin, more MVPA was associated with lower age, being employed, normal weight, and non-smoking, more LPA only with higher age, while singles, men, unemployed, and obese persons engaged in less LPA. Inactivity was associated with obesity and higher education.

**Conclusions:**

Singaporean participants engaged more in both MVPA and LPA than those from Berlin. Factors associated with PA varied considerably between both urban populations but also in relation to PA intensities. These variations highlight the need for tailored PA promotion strategies that distinguish between reducing inactivity and increasing overall activity levels.

**Supplementary Information:**

The online version contains supplementary material available at 10.1186/s12889-025-22922-x.

## Background

Physical activity (PA) is a crucial component in the prevention of non-communicable chronic diseases while inactivity is associated with, for example, cardiovascular diseases, type-2-diabetes, different types of cancer, and mortality [1–3]. While globally about one-third of the adult population does not meet the Global Recommendations on Physical Activity of the World Health Organisation (WHO) [4], there are large differences between countries [5].

In Germany, for example, approximately 42% of the population are not meeting the recommended levels of physical activity, based on population-based questionnaire data, while in Singapore, only around 20% of the population are not meeting the recommended activity guidelines, derived from population-based survey responses [6]. Reasons behind these cross-cultural differences in PA are multifaceted; even if both cities might be comparable in terms of income, education, or health care system, work-related factors, cultural norms, urban planning, access to recreational facilities, and the implementation of health campaigns can significantly influence an individual’s likelihood to engage in physical activity [7, 8]. While in Germany, for example, health promotion campaigns are often organized by local governments or communities [9, 10], the Singaporean government provides nationwide programs, campaigns, supporting materials (e.g. training videos), and the planning of sports facilities for the population through the ministry of health, the health promotion board, and other organization such as “Sport Singapore” or “Active Health” [11–14]. Also, Singapore and Berlin take very different approaches to urban design. Singapore’s centralized, master-planned strategy creates high-density, mixed-use areas that blend nature with urban life—a concept often called the “City in a Garden” [15]. In contrast, Berlin’s urban fabric evolved more organically, with a rich mix of historical architecture and a decentralized planning process that preserves cultural heritage while embracing modern development [16]. Understanding these and other cultural nuances is essential for tailoring effective public health strategies and interventions. Guided by the social determinants of health framework, our study is based on the premise that individual physical activity patterns are significantly influenced by key sociodemographic factors and health behaviors—such as education, age, sex, employment, marital status, BMI, and smoking. By comparing urban populations in Berlin and Singapore, we aim to elucidate how these determinants vary across contexts, thereby providing insights that can inform tailored public health strategies.

While recognizing the importance of physical activity in maintaining health, it’s crucial to acknowledge the limitations of using self-report questionnaires as the primary method for data collection [17]. Such questionnaires are not only subject to recall bias, social desirability bias, and inaccuracies, but can also lead to cultural differences in survey responding [18]. Regarding PA, several studies have reported discrepancies between self-reported and accelerometer measured physical activity, including an overestimation of moderate-to-vigorous physical activity (MVPA) and an underestimation of sedentary time when relying on self-reports [19–21]. The use of accelerometers allows for continuously measuring PA over a period of several days including daily duration and variations of PA intensities [22, 23].

Consequently, there has been a surge in the use of quantitative measurement of physical activity in recent years, such as accelerometers or pedometers [24]. Activity sensors have already been used in over 150 cohort studies including large-scale cohort studies like the UK Biobank, NHANES, or the 1970 British Cohort Study [17, 25–27]. Furthermore, there is an increasing demand in implementing device-measured physical activity in PA guidelines such as step counts or the use of consumer device measured activity [28, 29].

However, international data exchange in general and the harmonization of objectively measured PA data from cross-national cohorts in particular is still scarce, even if first steps are taken by proposing an International Activity Monitor Database [23, 30]. By harmonizing accelerometry and other essential data, cross-population comparisons can be performed regarding time spent in PA as well as different PA intensities, but also determinants associated with PA.

The aims of this study were therefore (i) to describe time spent in different intensities of PA in population-based studies from Singapore and Berlin by harmonizing accelerometer-measured, questionnaire-based, and medical examination data from different studies, and (ii) to investigate differences between associated factors including sociodemographic, and lifestyle factors in the two cohorts.

## Methods

### Study populations/ participants

This study used data from the Singapore Population Health Studies and the study center “Berlin-Mitte” of the German National Cohort (NAKO Gesundheitsstudie).

### Singapore population health studies (SPHS)

SPHS includes several prospective cohort studies. Data were used from the Singapore Health Study 2 (2014–2015) and from the Multi-Ethnic Cohort (MEC) (first follow-up of MEC phase 2) (2018–2019), which include randomly assigned hip-worn accelerometer assessments [31].

The Singapore Health 2 (SH2) is a nationally representative study including 2,686 Singapore residents aged 18 to 79 years, investigating physical, mental, and self-rated overall health, conducted between 2014 and 2015 [31]. Participants were invited to the study by contacting randomly selected households. Participants were interviewed and examined by trained study staff. The study was approved by the National University of Singapore Institutional Review Board (NUS IRB: reference 13–512). Written informed consent was obtained from all participants. For this study accelerometry data were used from the 895 subjects who participated in the physical activity sub-study [32, 33].

The Singapore Multi-Ethnic Cohort (MEC) is a prospective cohort study investigating environmental, lifestyle, and genetic determinants of non-communicable diseases [34, 35]. Data is assessed using standardized interviews and physical examinations. Multiple phases and follow-ups have been conducted, further detailed information about all phases of the MEC can be found on the study website [31]. For the present analyses, data were used from a sub-study of the first follow-up of MEC phase 2 (“Parks and Health project”) that was conducted between 2018 and 2019 [36]. It was approved by the National University of Singapore Institutional Review Board (B-16-125, 13–257). The “Parks and Health” project included 3,435 participants aged 21 to 75 years who received a home interview as well as a physical examination in a health screening centre including accelerometry in a randomly chosen sample of 500 participants [36].

### German National Cohort (NAKO)

NAKO is a nation-wide population-based prospective cohort study with more than 205,000 participants aged 20–69 years, conducted in 18 study centers in Germany [37, 38]. The aim of the study is to identify risk factors of chronic diseases such as cardiovascular and lung diseases, diabetes, cancer, neurodegenerative, or psychiatric diseases, and to provide data bases for the development of prevention measures. Data are assessed via standardized interviews, self-completed questionnaires, a variety of physical examinations, and collection of bio samples. Accelerometers are handed out to a random sample of 50% of all participants. Data collection is described in detail elsewhere [39]. In this study, data of the NAKO study center “Berlin-Mitte” were used, assessed at baseline between 2014 and 2019. Participants were drawn randomly from the Berlin registration office and invited to the study center. All participants gave their written informed consent. The study was approved by the ethical review committee of the Charité-Universitätsmedizin Berlin (EA1/076/13). For better comparison with the Singapore sample, recruitment periods were matched and therefore only participants recruited between 2014 and 2015 and 2018–2019 were included.

### Measures

#### Assessment and processing of the outcome physical activity

In all studies physical activity was objectively assessed by hip-worn accelerometers covering an activity range of ± 6 times the earth’s gravity (*g*) when monitoring the participants (GT3X/+, Fa. ActiGraph, Pensacola, FL, USA). Participants received the accelerometer at the study center where it was placed at the hip on the side of the dominant hand attached with an elastic belt. They were asked to wear it for one week, but they were allowed to take it off for showering or swimming (in Singapore also during the night). The device collected raw data at a sampling rate of 100 Hz in the Berlin sample and of 30 Hz in the Singapore samples. The raw gtx3 accelerometry data collected by the devices was processed into csv files using ActiLife version 6.11.9 [40]. Minimum wear criteria for an evaluated day to be considered a valid day was set to 12 h in contrast to many other studies using 10 h [41, 42]. This stricter criterion helps to capture variability across the entire waking day and improves the reliability and comparability of the derived activity measures. The evaluated times of movement were set between 6 am and 11 pm for both NAKO and Singapore data. Data from participants who provided fewer than two valid weekdays as well as at least one weekend day were excluded. Processing of the tri-axial accelerometry data from the participants was done with the R-package multi-step procedure of GGiR Version 2.5-0 [43, 44]. To compensate for calibration errors of the accelerometry device, the protocol of GGiRs auto-calibration was followed, which computes the devices off set and adjusts the acceleratory data accordingly [44]. The calibrated data was then used for the classification of the different intensity levels of PA. A commonly chosen approach is using the Euclidean Norm Minus One (ENMO) [45]. This metric utilizes the acceleration vector’s magnitude while accounting for the effects of gravity to summarise the tri-axial data into one value [46]. GGiR computes the magnitude by default for intervals of 5 s. With the computation of this metric, GGiR can classify the data into intensity levels of PA. We set the upper-class boundaries for inactivity (defined as periods with minimal or no detectable movement), and the three PA intensities light, moderate, and vigorous PA to ENMO = 10, 69, 258, and 10,000 mg, respectively, as proposed by earlier studies evaluating accelerometer-measured PA intensity thresholds [47, 48].

#### Sociodemographics

Sociodemographic variables were harmonized across studies by matching respective variable categories. The variables age (in years), sex (male/female), marital status (single, married, divorced/widowed), and employment status (‘employed’, ‘not employed’, and ‘retired/not able to work’) were assessed in a comparable manner in both samples. Education was transformed according to ISCED-97 guidelines into the classification ‘low’, ‘middle’, and ‘high’, household net-income was not comparable between samples and was therefore categorized into ‘above average’, ‘average’, ‘below average’ for each sample respectively. Ethnicity (for the Singapore sample) was categorized into ‘Chinese ethnicity’ vs. ‘other than Chinese’, and migration background (for the Berlin sample) was categorized into ‘migration background’ and ‘no migration background’; migration background was defined as having (i) own migration experience (without German nationality) or (ii) as having at least one parent with own migration experience [49]. For the pooled analysis, a new variable ‘migration reference’ was created to include ethnicity and migration background.

#### Health behaviors and diseases

In the NAKO, trained study personnel measured body height to the nearest 0.1 cm and body weight to the nearest 0.1 kg using a calibrated integrated measurement station (SECA model 764, Seca^®^, Hamburg, Germany). In the Singapore studies, weight and height were assessed via self-report. Body mass index (BMI) was calculated from these measurements/self-reports as weight over height squared in kg/m², and categorized into underweight (BMI < 18.5 kg/m²), normal weight (BMI 18.5–24.9 kg/m² (Berlin); 18.5–22.9 kg/m² (Singapore)), overweight (BMI 25.0–29.9 kg/m² (Berlin); BMI 23.0–27.4 kg/m² (Singapore)), and obesity (BMI > 30.0 kg/m² (Berlin); BMI > 27.5 kg/m² (Singapore)) using Asian cut-offs for the Singapore sample, as it has been shown to be appropriate to lower BMI cut-offs for Asians due to higher metabolic and cardiovascular risk already at a lower BMI than in Caucasians [50–53]. Smoking status was categorized into smokers (regular smoking), ex-smokers, and never-smokers. Risk factors and diseases were assessed via self-report of physician’s diagnosis, including lifetime prevalence of hypertension, diabetes, dyslipidemia, cardiovascular diseases (angina pectoris, heart attack, heart failure, stroke, or arrhythmia), lung diseases (asthma, chronic obstructive pulmonary disease (COPD)), and cancer. All diseases were transformed into a dichotomous variable ‘at least one chronic disease’.

### Statistical analysis

The present analyses are based on an exploratory approach. Participants’ sociodemographic and health behavior characteristics were analyzed using descriptive methods of means and standard deviations (SD) for continuous data and absolute and relative frequencies for categorical data. Missing data were not imputed. To avoid data loss, a binary ‘missing’ category was added to the variable with the highest number of missings (‘education status’ for the Berlin sample and ‘income’ for the Singapore sample) and included in the regression analyses (see Supplementary tables). Multivariable linear regression analyses were used to examine associations of sociodemographics and lifestyle with all PA intensities (inactivity, light, and moderate-to-vigorous PA), results are presented as average mean differences of PA minutes. In our regression models, we included a full set of sociodemographic and health-related covariates to estimate the independent association of each factor with PA and inactivity. As such, the estimates presented reflect direct effects. All analyses were performed separately for Singapore and Berlin as well as for the combined cohorts. All statistical analyses were done using Python Version 3.9.7, and the regression models of Statsmodels Version 0.13.2 [54].

## Results

### Participants

**Fig. 1:**
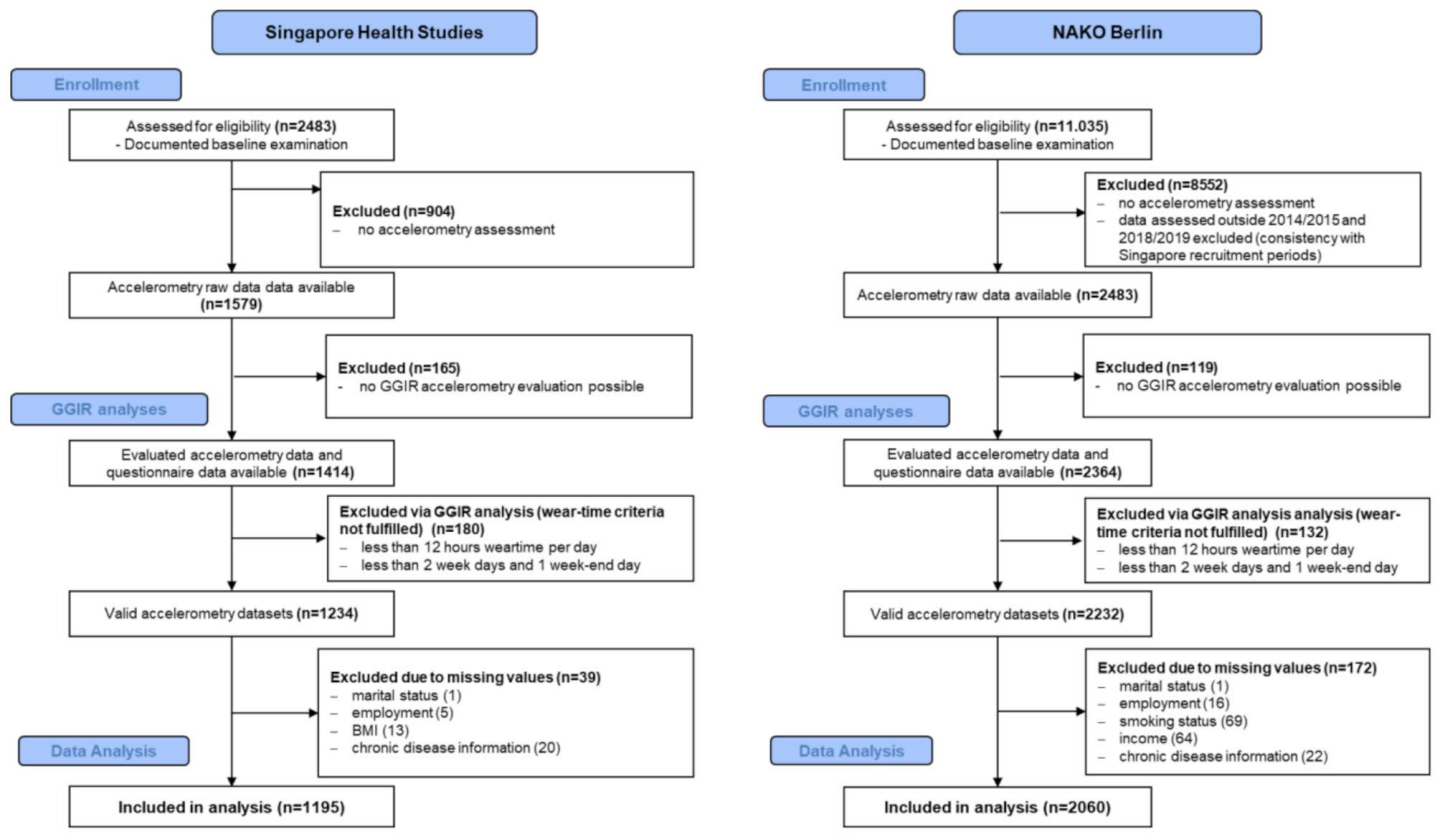
Enrolment flowcharts for Singapore and Berlin (exclusion criteria are described in more detail in the methods section)

In Berlin, 11,035 individuals (with a 17% response rate) participated in the NAKO baseline examination. After excluding those examined outside the 2014–2015 and 2018–2019 periods and participants who did not receive an accelerometer, 2,483 individuals were eligible for our analysis. Of these, 2,060 (83.0%) were included in the final analysis after applying wear time validation. For the Singapore cohort, out of 2,483 participants of the Singapore Health Studies, 1,579 participants wore an accelerometer, and 1,195 (75.7%) datasets could be included after applying wear time validation (Fig. 1).

Participants of the Singapore and Berlin cohort exhibited differences in key demographic characteristics. Notably, participants of the Singapore cohorts were younger, were more often married, and were more often non-smokers. Moreover, fewer participants were categorized as having high or moderate compared to low educational level, fewer participants were retired compared to the Berlin cohort. Further details are displayed in Table 1.

**Table 1:** Participant characteristics in Singapore and Berlin

Sociodemographic and clinical characteristics	Singapore (*n* = 1,195)	Berlin (*n* = 2,060)
*Sociodemographics*	*n* (%) or mean ± SD	
Women	683 (57.2%)	1,015 (49.3%)
Age (years)	46.6 ± 13.7	49.9 ± 12.6
Marital status		
Married	790 (66.1%)	1,020 (49.5%)
Single	288 (24.1%)	725 (35.2%)
Divorced/widowed	117 (9.8%)	315 (15.3%)
Education level (ISCED 97^*^)		
High	690 (57.7%)	1,248 (60.6%)
Middle	214 (17.9%)	525 (25.5%)
Low	291 (24.4%)	38 (1.8%)
Missing (applies only to Berlin)	-	249 (12.1%)
Migration background (applies only to Berlin)	-	335 (16.3%)
Ethnicity (applies only to Singapore)		-
Chinese	841 (70.4%)	-
Malay	147 (12.3%)	-
Indian	142 (11.9%)	-
others	65 (5.4%)	-
Employment status		
Employed	889 (74.4%)	1,579 (76.7%)
Not employed (student, house maker)	211 (17.7%)	102 (5.0%)
Retired, not able to work	95 (8.0%)	379 (18.4%)
Monthly net household income		
Above sample average	387 (32.4%)	731 (35.5%)
Sample average	240 (20.1%)	460 (22.3%)
Below sample average	480 (40.2%)	869 (42.2%)
Missing (applies only for Singapore)	88 (7.4%)	-
*Health behaviours*		
BMI in kg/m²**	24.6 ± 4.7	25.7 ± 4.5
Underweight	59 (4.9%)	33 (1.6%)
Normal weight	418 (35.0%)	984 (47.8%)
Overweight	467 (39.1%)	725 (35.2%)
Obesity	251 (21.0%)	318 (15.4%)
Smoking behaviour		
Regular smoker	148 (12.4%)	478 (23.2%)
Ex-smoker	99 (8.3%)	681 (33.1%)
Never-smoker	948 (79.3%)	901 (43.7%)
*Clinical condition*		
At least one chronic disease*** (ever diagnosed by physician)	518 (43.4%)	1,039 (50.4%)

*ISCED = International Standard Classification of Education 97. **BMI (body mass index) based on measured weight and height in the Berlin sample and on self-report in the Singapore sample. BMI cut-offs are different for both samples according to general and Asian-specific cut-offs proposed by the WHO expert consultation [50, 51]. ***Chronic diseases include: metabolic diseases, (hypercholesterolemia, diabetes), cardiovascular diseases (hypertension, angina pectoris, heart attack, stroke, heart failure, arrhythmia), cancer, lung diseases (asthma, COPD)

### Accelerometer-measured physical activity and inactivity

Mean number of days with valid wear time were 6.6 ± 0.8 days in Singapore (4.7 ± 0.8 weekdays and 1.9 ± 0.5 weekend days) and 5.8 ± 0.6 days in Berlin (3.9 ± 0.5 weekdays and 1.9 ± 0.3 weekend days). Singapore participants spent more time of the day in MVPA (8.3 vs. 6.8%) and LPA (32.2 vs. 23.7%) and less time in inactivity (59.5 vs. 69.5%) than Berlin participants. Compared to men, Singaporean women tended to have lower levels of LPA and MVPA, and higher levels of inactivity. In contrast, men and women in Berlin had similar levels of MVPA and inactivity, while women spent a little more time in LPA. (Table 2). More information regarding MPA, VPA, and bouted MVPA are shown in Supplementary Table 3. Further differences in time spent in different PA intensities and inactivity according to participant characteristics were observed in Singapore and Berlin (Supplementary Fig. 1).

**Table 2:** Accelerometer-measured physical activity (PA) and inactivity

Variables	Singapore			Berlin		
	Total (*n* = 1,195)	Women (*n* = 683)	Men (*n* = 512)	Total (*n* = 2,060)	Women (*n* = 1,015)	Men (*n* = 1,045)
	*n* (%) or mean ± SD					
**Wear time**						
Number of valid days	6.6 ± 0.8	6.6 ± 0.8	6.6 ± 0.8	5.8 ± 0.6	5.9 ± 0.5	5.8 ± 0.6
Weekdays	4.7 ± 0.8	4.7 ± 0.8	4.7 ± 0.8	3.9 ± 0.5	3.9 ± 0.5	3.9 ± 0.5
Weekend-days	1.9 ± 0.5	1.9 ± 0.5	1.9 ± 0.5	1.9 ± 0.3	1.9 ± 0.3	1.9 ± 0.3
Average wear time per day (minutes)	807.6 ± 98.8	811.9 ± 96.5	802.0 ± 102.2	848.2 ± 78.7	853.3 ± 73.2	843.2 ± 83.5
**Types of activities in** **minutes per day**						
Moderate-to-vigorous PA	66.6 ± 55.8	63.1 ± 55.5	71.2 ± 55.9	58.0 ± 26.0	58.0 ± 25.4	58.1 ± 26.4
Light PA	259.1 ± 132.0	250.6 ± 128.6	270.4 ± 135.6	201.4 ± 78.8	206.0 ± 79.9	196.8 ± 77.4
Inactivity	482.0 ± 161.6	498.2 ± 155.9	460.3 ± 166.6	588.8 ± 95.1	589.2 ± 93.3	588.3 ± 96.9
**Types of activities in** **percent per day**						
Moderate-to-vigorous PA	8.3 ± 6.7	7.8 ± 6.8	8.9 ± 6.5	6.8 ± 2.9	6.8 ± 2.9	6.9 ± 3.0
Light PA	32.2 ± 16.2	30.9 ± 15.4	33.9 ± 16.9	23.7 ± 9.0	24.1 ± 9.0	23.3 ± 9.0
Inactivity	59.5 ± 18.4	61.3 ± 17.5	57.2 ± 19.2	69.5 ± 9.9	69.2 ± 9.9	69.9 ± 10.0

### Physical activity during the week

Also on a daily basis, PA levels were higher in Singapore than in Berlin. In Singapore, MVPA, LPA and inactivity were relatively stable throughout the week with a small decline of MVPA on week-end days.

**Fig. 2:**
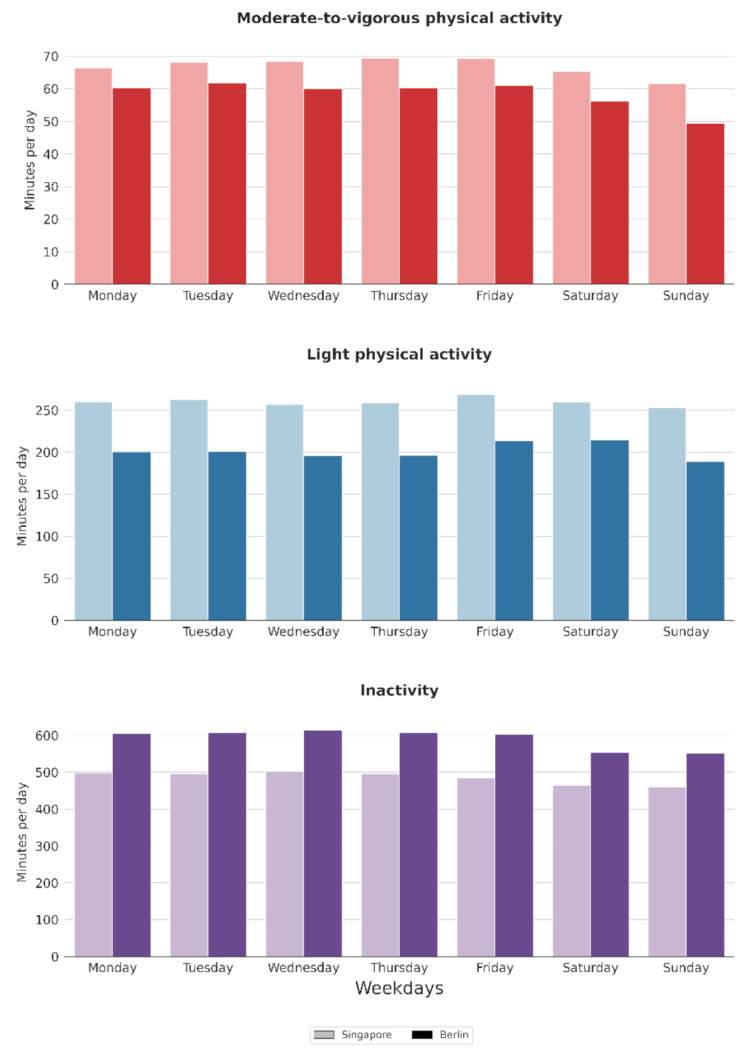
Weekly minutes of moderate-to-vigorous and light physical activity, and of inactivity in Singapore (lighter bars) and Berlin (darker bars)

In Berlin, the pattern was very similar for MVPA. LPA, however, peaked on Friday and Saturday, while inactivity decreased on these days (Fig. 2). For the percentage distribution over the week see supplementary Table 4.

### Factors associated with physical activity intensities and inactivity in Singapore and Berlin

The pooled multivariable regression analysis showed that Singapore participants spent significantly more time in MVPA and LPA and less time in inactivity than Berlin participants (Table 3). Two factors were associated with MVPA, LPA, and inactivity, namely, body weight and unemployment. Both higher body weight and unemployment were linked to less MVPA and LPA, but to more inactivity. Older age and lower education were inconsistently associated with PA (less MVPA but more LPA, with no association with inactivity). Marital status was only associated with LPA and inactivity; married individuals exhibited more LPA and less inactivity than singles, divorced, or widowed persons. For more details see supplementary Figs. 2–4.

**Table 3:** Multivariable regression analysis for the outcomes of moderate-to-vigorous physical activity, light physical activity, and inactivity

Variables	Moderate-to-vigorous physical activity		Light physical activity		Inactivity	
	Mean difference [95% CI]	*p*-value	Mean difference [95% CI]	*p*-value	Mean difference [95% CI]	*p*-value
**Study sample (ref: Berlin)**						
Singapore	14.1 [10.6;17.5]	< 0.01	66.2 [57.4;74.9]	< 0.01	-80.2 [-90.0;-70.4]	< 0.01

The model was adjusted for age, sex, marital status, education level, migration background/ethnicity, employment status, income, BMI, smoking behavior, clinical condition, and wear time; CI: confidence interval

**Fig. 3:**
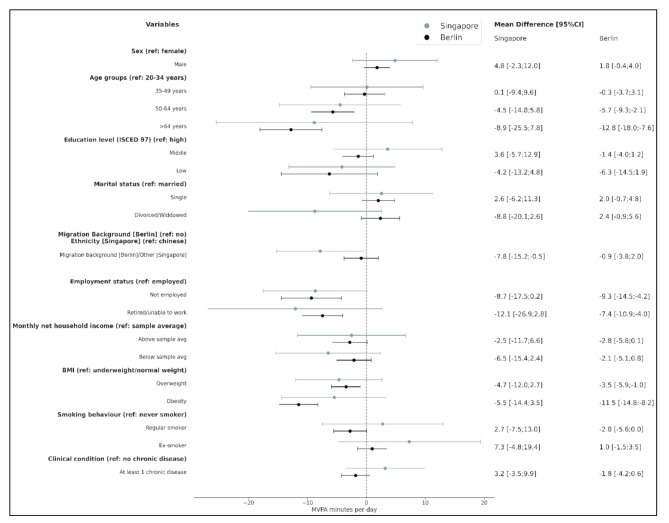
Multivariable regression analyses (mutually adjusted for all covariates) for Singapore and Berlin. **Outcome: Moderate-to-Vigorous Physical Activity (MVPA)**-minutes per day. The coefficient represents mean differences in MVPA minutes with 95% confidence intervals (95%CI). (Note: BMI categories differ by cohort following WHO recommendations (Asia-Pacific cut-offs for Singapore: normal weight: BMI 18.5–22.9 kg/m², overweight: 23.0–27.4 kg/m², obesity: >27.5 kg/m²) and standard WHO cut-offs for Berlin: normal weight: BMI 18.5–24.9 kg/m², overweight 25.0–29.9 kg/m², and obesity > 30.0 kg/m²); direct cross-cohort comparisons should therefore be interpreted with caution.)

**Fig. 4:**
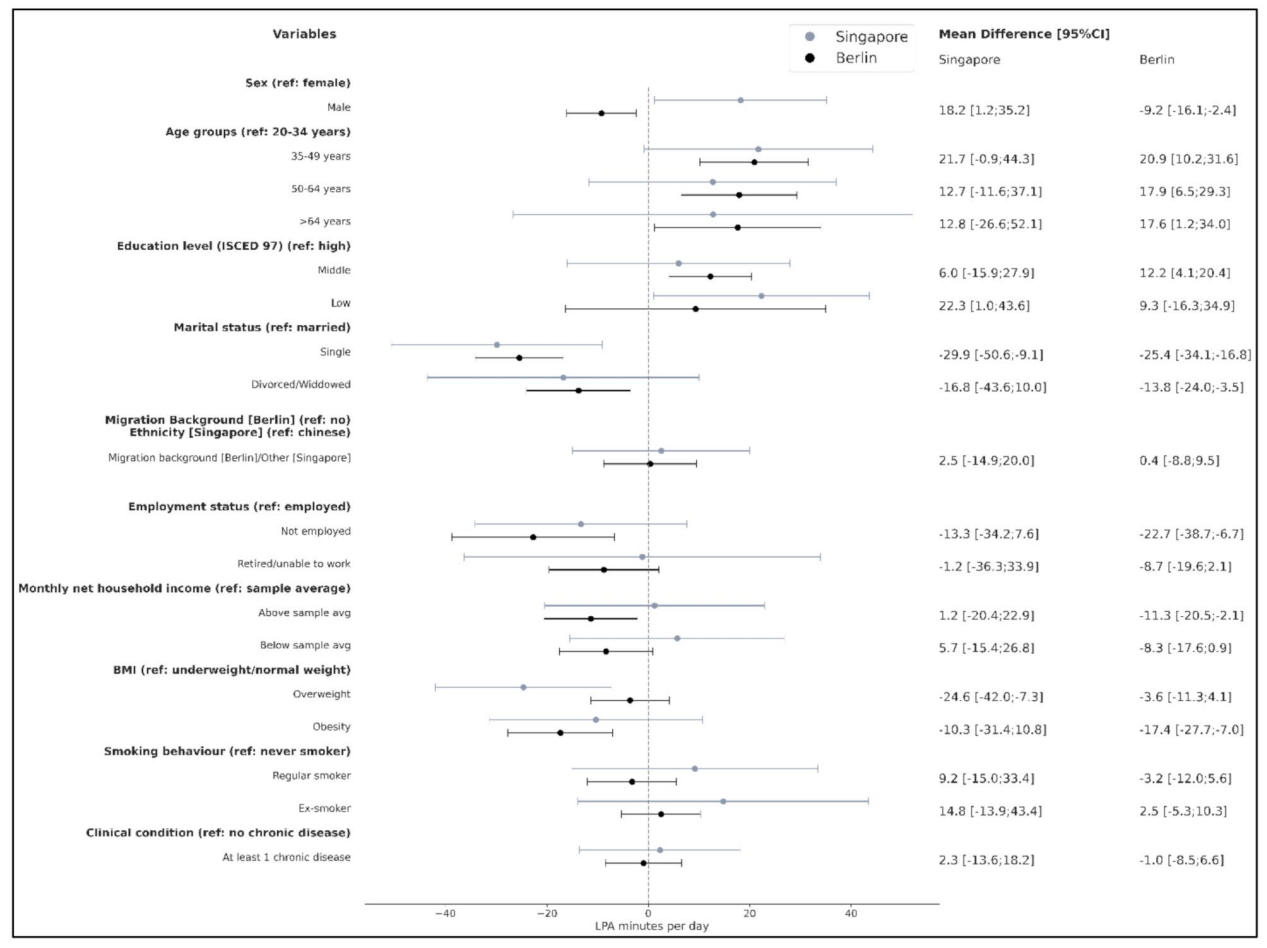
Multivariable regression analyses (mutually adjusted for all covariates) for Singapore and Berlin. **Outcome: Light Physical Activity** (LPA)-minutes per day. The coefficient represents mean differences in LPA minutes with 95% confidence intervals (95%CI). (Note: BMI categories differ by cohort following WHO recommendations (Asia-Pacific cut-offs for Singapore: normal weight: BMI 18.5–22.9 kg/m², overweight: 23.0–27.4 kg/m², obesity: >27.5 kg/m²) and standard WHO cut-offs for Berlin: normal weight: BMI 18.5–24.9 kg/m², overweight 25.0–29.9 kg/m², and obesity > 30.0 kg/m²); direct cross-cohort comparisons should therefore be interpreted with caution.)

**Fig. 5:**
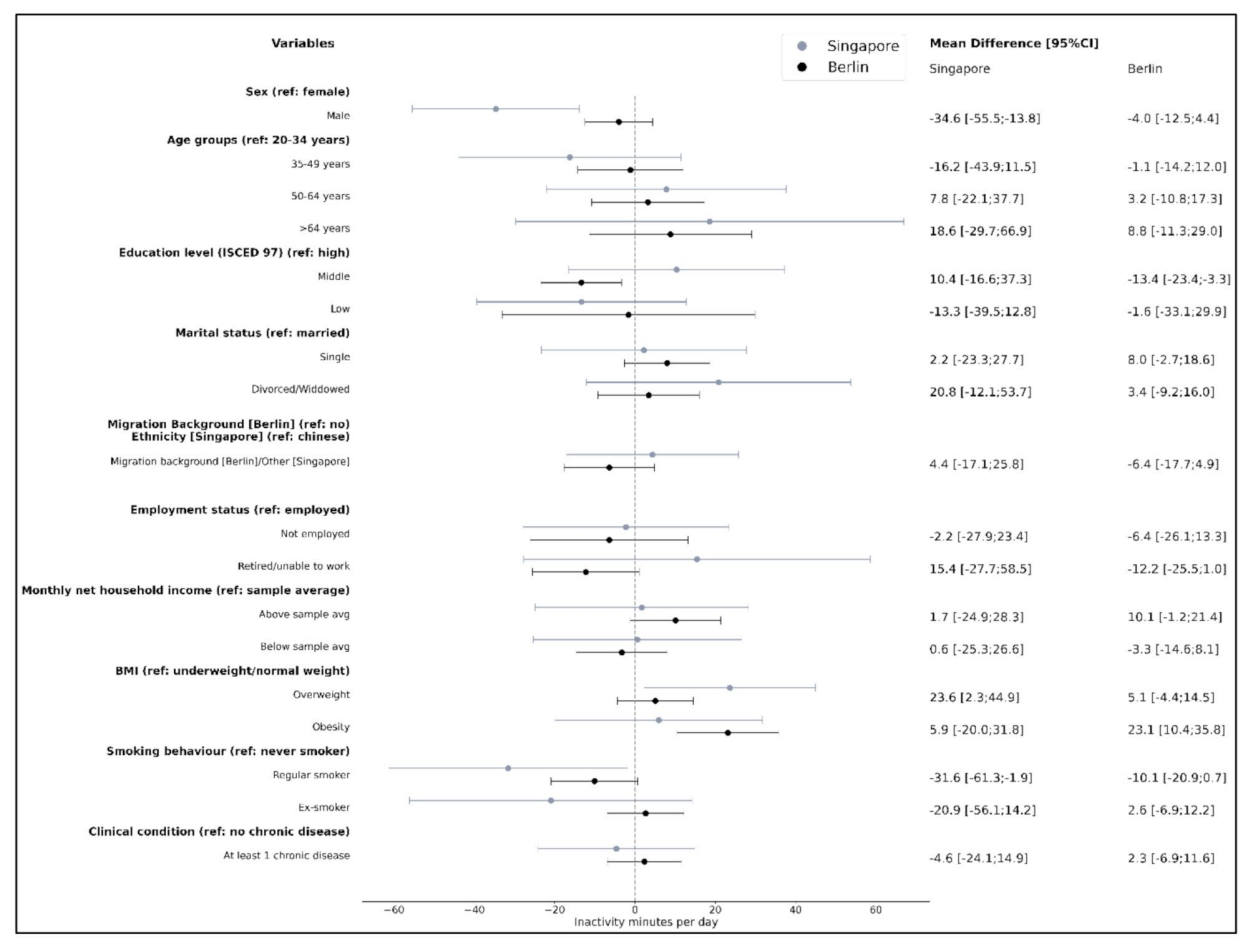
Multivariable regression analyses (mutually adjusted for all covariates) for Singapore and Berlin. **Outcome: Inactivity** minutes per day. The coefficient represents mean differences in inactivity minutes with 95% confidence intervals (95%CI). (Note: BMI categories differ by cohort following WHO recommendations (Asia-Pacific cut-offs for Singapore: normal weight: BMI 18.5–22.9 kg/m², overweight: 23.0–27.4 kg/m², obesity: >27.5 kg/m²) and standard WHO cut-offs for Berlin: normal weight: BMI 18.5–24.9 kg/m², overweight 25.0–29.9 kg/m², and obesity > 30.0 kg/m²); direct cross-cohort comparisons should therefore be interpreted with caution.)

Separate multivariable regression analyses for Singapore and Berlin suggest that among Singapore participants, MVPA was negatively associated with other than Chinese ethnicities (-7.8 [-15.2;-0.5]), and with unemployment (-8.7 [-17.5;0.2]; borderline significant) (Fig. 3–5).

Men (18.2 [1.2;35.2]) and participants with low education (22.3 [1.0;43.6]) spent more time in LPA than women, and participants with high education, respectively, while being single (-29.9 [-50.6;-9.1]) and being overweight (-24.6 [-42.0;-7.3]) was associated with less LPA minutes (Fig. 3–5).

More inactivity was associated with overweight (23.6 [2.3;44.9]) compared to normal weight, while men (-34.6 [-55.5;-13.8]) and regular smokers (-31.6 [-61.3;-1.9]) spent less time in inactivity (Fig. 5).

Among Berlin participants, more MVPA was reversely associated with higher age (-5.7 [-9.3;-2.1] in age group 50–64, and − 12.8 [-18.0;-7.6] in age group 65 + compared to age group 20–34 years), with unemployment (-9.3 [-14.5;-4.2]) or retirement (-7.4 [-10.9;-4.0], and with overweight (-3.5 [-5.9;-1.0]) and obesity (-11.5 [-14.8;-8.2]), a trend was seen for the association with male sex (1.8 [-0.4;4.0]) (Fig. 3).

Higher age was associated with more LPA minutes among all age groups (35–49: 20.9 [10.2;31.6], 50–64: 17.9 [6.5;29.3], and 65+: 17.6 [1.2;34.0], respectively, compared to age group 20–34 years), and with having a middle compared to high education (12.2 [4.1;20.4], while male sex (-9.2 [-16.1;-2.4]), being single (-25.4 [-34.1;-16.8]) or divorced/widowed (-13.8 [-24.0;-3.5]), not being employed (-22.7 [-38.7;-6.7]), having an income above (-11.3 [-20.5;-2.1]) or below (-8.3 [-17.6;0.9]; trend) the average income as well as obesity (-17.4 [-27.7;-7.0]) were associated with lower LPA (Fig. 4).

Inactivity was associated with obesity compared to normal weight (23.1 [10.4;35.8]). Less time in inactivity was associated as a trend with high income (10.1 [-1.2;21.4]), middle compared to high educational level (-13.4 [-23.4;-3.3]), retirement (-12.2 [-25.5;1.0]), and regular smokers (-10.1 [-20.9;0.7]) spent less time in inactivity (Fig. 5). All results are displayed in table format including supplemental analyses (moderate PA, vigorous PA) in Supplementary Table 1 (Singapore) and Supplementary Table 2 (Berlin).

## Discussion

### Main findings

#### Comparison of Singapore and Berlin in the context of other studies

In Singapore, slightly more MVPA and considerably more LPA was observed than in Berlin, irrespectively of confounding factors such as age, body weight, socioeconomic or lifestyle factors. Although it is not possible to quantify the health effect due to the cross-sectional design of our study, research investigating dose-response associations between PA and health outcomes led to high evidence that an increase of any intensity and duration of PA is associated with a reduction of mortality [30, 55]. While both cities are highly urbanized, with infrastructure promoting active lifestyles, namely parks, sports facilities, and public transport, there seem to be differences that lead to this activity gap. One important reason seem to be the various governmental activities including nationwide health campaigns such as the “war on diabetes” campaign or the “step challenge”, the creation of sports facilities, and a broad range of sporting opportunities for all groups of the population [11, 13, 14, 56–58]. The urban structure of the cities may be another explanation for differences in PA. Although Singapore and Berlin both focus on expanding and maintaining their parks, it seems these as well as other innovative infrastructure are more meticulously planned and easier accessible within the city core of Singapore in contrast to the more historically grown city of Berlin, which could further contribute to this difference in PA [59, 60]. Another possible factor is Singapore’s consistent climate, which may encourage more LPA due to the feasibility of walking outdoors more frequently compared to Berlin’s varied climate [61, 62]. On the other hand, the tropical climate may reduce more intense activities. This may be reflected in the sports preferences with walking as the preferred sports in Singapore, and cycling in Berlin [7, 8]. However, a recent study from Singapore showed that for many people acclimatized to the tropics, the climate is a less important barrier than factors such as safety and convenience [63]. Interpreting these findings through the social determinants of health framework further illuminates the interplay between individual behavior and broader contextual factors. This framework posits that health behaviors, including physical activity, are shaped not only by personal choices but also by socioeconomic, cultural, and environmental conditions. In our study, differences in urban planning, governmental initiatives, and climatic conditions between Singapore and Berlin appear to contribute to the observed activity gap, while individual-level determinants—such as ethnicity, employment status, and body weight—interact with these structural factors and are associated with physical activity patterns. Finally, inaccurately capturing the activity level during cycling might influence the outcome of MVPA to the disadvantage of Berlin participants, although the overall impact may be small [64].

#### Associated factors with MVPA

It has been well described that MVPA is inversely related to age [65]; this trend is also displayed in both cohorts, while only significant in the Berlin population. Additionally, males have been shown to engage in more MVPA than women [66, 67]. This was also the case in the Singapore sample, while the difference was smaller in Berlin. Reasons may be rather cultural than environmental since the infrastructure in Singapore is very sports promoting. On the other hand, the correlation between BMI and MVPA, which has consistently been described in the literature [68] was significant in the Berlin population, while in Singapore this was seen only as a trend. The proactive health campaigns in Singapore enhancing all persons, indiscriminatory of BMI-groups to be more inclined to participate in PA might be one reason for this observation [69]. Regarding employment status, some evidence points out the association between higher PA in working persons both on weekdays and weekend days, underscoring our observation of reduced MVPA for retired and unemployed persons in both samples [70]. The difference between working and non-working persons is also supported by the work of Pulakka et al. except that their differences arise only from weekday PA [71]. Moreover, active transport could be a leading factor in the differences in PA between working and non-working persons, given their commute [72]. Furthermore, Chinese ethnicity in the Singaporean cohort was a significant indicator for MVPA. This result is in line with other studies [33, 73]. Given, Malays and Indians have a higher risk of obesity, this might be a mediator for the engagement in PA [34].

#### Associated factors with LPA

The results regarding the association of sex with LPA were contradictory in the two cohorts with Singaporean men engaging in more whilst Berlin men engaging in less LPA than women. Underlying reasons might be cultural aspects, or differences in active transport (less Singaporean women than men reported being employed) [70]. Both cohorts display an association with fewer minutes of LPA and overweight (Singapore) and obesity (Berlin), which is in line with previous research in German and Singaporean populations [74]. Likewise, singles spent less time in LPA than married persons. There is little evidence for the influence of marital status on PA, while heterogenous reports propose single persons spend more time in MVPA and married persons engage more in household-related movements [75, 76].

#### Associated factors with inactivity

There were only few factors associated with inactivity. Singaporean males were less inactive than women, while there was no such difference in the Berlin sample. Underlying reasons might be cultural factors, working behaviour, or different coping mechanisms for the tropical climate. The higher amount of time spent by overweight and/or obese persons in inactivity is well described in the existing literature and in accordance with research in cross-sectional and longitudinal studies [77–79]. Both cohorts also exhibited less inactivity for smokers. This might be explained by the place change association to have a smoke. A German study has shown this association, while in Singapore, no population study is available, supposably because smoking prevalence is very low overall in Singapore [74].

### Strengths and limitations

The major strength of this study is the large number of participants enrolled in the two population-based cohorts, along with their harmonized data. To our knowledge, this is the first study to publish harmonized accelerometry data from two urban areas in Europe and Asia. Secondly, PA was measured with accelerometers, providing precise and continuous measurement of PA data of week- and weekend days, avoiding bias introduced through self-reported PA. Thirdly, Singapore and Berlin provided extensive data on sociodemographic, lifestyle behavior and prevalent chronic diseases through which it was possible to investigate associations with PA.

There are also some limitations. Firstly, there were differences in wear protocol between the cohorts, which could have introduced unwanted biases in the distribution of PA throughout the captured day. While not completely eliminating wear time bias, it was mitigated by restricting accelerometer analysis to the period between 6 am and 11 pm. Additionally, using a minimum wear time of 12 h instead of more often used 10 h reduces the risk of overestimating physical activity levels due to partial-day recordings and ensures a more comprehensive representation of daily activity patterns [41, 42]. Another limitation is that PA was collected at 100 Hz in the Berlin sample, whereas it was collected at 30 Hz in the Singapore sample. While variation in sampling frequency can theoretically impact physical activity estimates, recent empirical research indicates that raw acceleration-based metrics, such as the Euclidean Norm Minus One (ENMO) used in our study, are highly robust across different frequencies. Clevenger et al. demonstrated statistical equivalence of raw-metric outcomes across sampling rates ranging from 30 to 100 Hz in a large free-living sample, with mean differences in moderate-to-vigorous physical activity typically under 3 min/day [80]. Similarly, Migueles et al. emphasize that while sampling frequency is a key methodological decision, its influence on raw acceleration data is minimal when harmonized processing protocols are applied [81]. In our analysis, we processed all raw data into a common csv format using ActiLife software and then applied a standardized, multi-step procedure with the GGIR R-package, which included auto-calibration to adjust for any device offsets. This harmonized approach mitigates potential discrepancies introduced by initial sampling differences, supporting the validity of our cross-cohort comparisons. Further, representativity of the samples is not guaranteed due to low response rates (especially in Berlin with only 17%). This could have introduced a selection bias with higher participation of rather healthy and active persons. Additionally, the distribution of sex, age, marital status, education level, smoking, and further lifestyle behaviors varied between the cohorts. Therefore, evaluating the factors’ association with PA might have varied with a larger sample size, which was counter-acted by including all variables in our full sample analysis. Due to the smaller size of the Singapore sample, our estimates there were less precise—evidenced by wider confidence intervals—which may obscure or distort the observed associations with physical activity compared to the Berlin cohort. Furthermore, hip-worn accelerometers may underestimate activities of the upper body and cannot detect whether a person is carrying any weight (e.g. grocery shopping). Finally, the cross-sectional design of our study does not allow causal inferences nor provides information on temporal associations between the associated factors and the different PA intensities. However, a follow-up analysis is planned as soon as follow-up data of cohorts will be available. With these long-term data, research gaps regarding dose-response associations, time-related changes and potential causal relationships between risk factors and the amount of different PA intensities could be addressed.

## Conclusion

Singapore participants engaged more often in both MVPA and LPA than Berlin participants who spent relatively more time in inactivity. Factors associated with PA differed between the populations and within the populations regarding PA intensities. The results might help to tailor PA promotion programs (e.g. community-based exercise programs, active transport infrastructure, workplace PA initiatives, and accessible recreational spaces) for different population groups within two culturally diverse populations.

## Electronic supplementary material

Below is the link to the electronic supplementary material.

Supplementary Material 1

## Data Availability

No datasets were generated or analysed during the current study.

## Abbreviations

BMI: Body mass index
CI: Confidence interval
ENMO: Euclidean norm minus one
G: Gravity
ISCED-97: International Standard Classification of Education 97
LPA: Light physical activity
MEC: The Singapore Multi-Ethnic Cohort
MPA: Moderate physical activity
MVPA: Moderate-to-vigorous physical activity
NAKO: National Cohort Study
PA: Physical activity
SD: Standard deviation
SH2: Singapore Health 2
SPHS: Singapore Population Health Studies
VPA: Vigorous physical activity
WHO: World Health Organization
