# Supplementary material for: Patterns and associated factors of accelerometer-measured physical activity in the metropolitan areas of Singapore and Berlin – comparative analysis of the Singapore population health studies and the German National Cohort (NAKO)
Source: BMC Public Health. 2025 May 21;25:1872. doi: 10.1186/s12889-025-22922-x (PMC12093802; doi:10.1186/s12889-025-22922-x)
Supplement: Supplementary file 1 — Supplementary Material 1 [file 12889_2025_22922_MOESM1_ESM.docx]

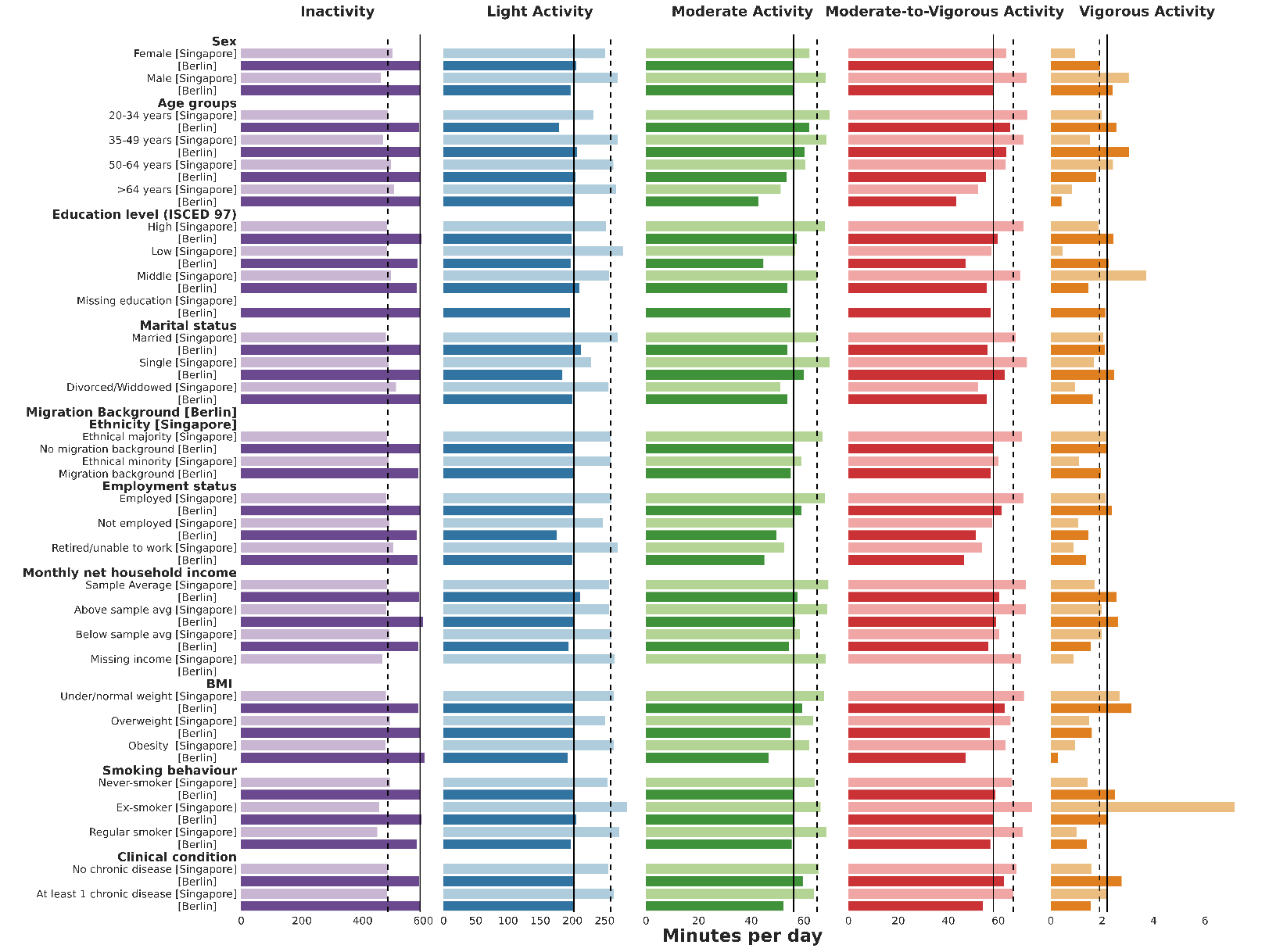


**Supplementary Figure 1:** Physical activity intensities stratified for sociodemographic factors, lifestyle behavior and clinical condition for Singapore (light colors) and Berlin (dark colors).

**Supplementary Table 1.** Adjusted associations of sociodemographic and health-related factors with inactivity, light physical acitivity (LPA), moderate physical activity (MPA), moderate-to-vigorous physical activity (MVPA), and vigorous physical activity (VPA) among the **Singaporean** sample.

| **Variables** | **Inactivity** | | **LPA** | | **MPA** | | **MVPA** | | **VPA** | |
| --- | --- | --- | --- | --- | --- | --- | --- | --- | --- | --- |
|  | minutes per day | | minutes per day | | minutes per day | | minutes per day | | minutes per day | |
|  | Mean difference [95% CI] | p-value | Mean difference [95% CI] | p-value | Mean difference [95% CI] | p-value | Mean difference [95% CI] | p-value | Mean difference [95% CI] | p-value |
| **Sex (ref: female)** |  |  |  |  |  |  |  |  |  |  |
| male | -34.6 [-55.5;-13.8] | <0.01 | 18.2 [1.2;35.2] | 0.04 | 3.3 [-3.3;9.9] | 0.32 | 4.8 [-2.3;12.0] | 0.19 | 1.5 [-0.5;3.6] | 0.14 |
| **Age groups (ref: 20-34 years)** | |  |  |  |  |  |  |  |  |  |
| 35-49 years | -16.2 [-43.9;11.5] | 0.25 | 21.7 [-0.9;44.3] | 0.06 | 0.9 [-7.9;9.6] | 0.85 | 0.1 [-9.4;9.6] | 0.99 | -0.8 [-3.4;1.9] | 0.58 |
| 50-64 years | 7.8 [-22.1;37.7] | 0.61 | 12.7 [-11.6;37.1] | 0.31 | -4.8 [-14.2;4.7] | 0.32 | -4.5 [-14.8;5.8] | 0.39 | 0.3 [-2.6;3.2] | 0.83 |
| >64 years | 18.6 [-29.7;66.9] | 0.45 | 12.8 [-26.6;52.1] | 0.52 | -8.7 [-23.9;6.5] | 0.26 | -8.9 [-25.5;7.8] | 0.30 | -0.1 [-4.8;4.5] | 0.95 |
| **Education level (ISCED 97) (ref: high)** | | | |  |  |  |  |  | |  |
| Middle | 10.4 [-16.6;37.3] | 0.45 | 6.0 [-15.9;27.9] | 0.59 | 1.8 [-6.7;10.3] | 0.68 | 3.6 [-5.7;12.9] | 0.45 | 1.8 [-0.8;4.4] | 0.17 |
| Low | -13.3 [-39.5;12.8] | 0.32 | 22.3 [1.0;43.6] | 0.04 | -2.7 [-11.0;5.5] | 0.52 | -4.2 [-13.2;4.8] | 0.37 | -1.4 [-4.0;1.1] | 0.27 |
| **Marital status (ref: married)** | | |  |  |  |  |  |  |  |  |
| Single | 2.2 [-23.3;27.7] | 0.87 | -29.9 [-50.6;-9.1] | <0.01 | 3.5 [-4.5;11.6] | 0.39 | 2.6 [-6.2;11.3] | 0.57 | -1.0 [-3.4;1.5] | 0.44 |
| Divorced/widdowed | 20.8 [-12.1;53.7] | 0.21 | -16.8 [-43.6;10.0] | 0.22 | -8.1 [-18.5;2.2] | 0.12 | -8.8 [-20.1;2.6] | 0.13 | -0.6 [-3.8;2.6] | 0.71 |
| **Ethnicity (ref: Chinese)** | |  |  |  |  |  |  |  |  |  |
| Other than Chinese | 4.4 [-17.1;25.8] | 0.69 | 2.5 [-14.9;20.0] | 0.77 | -7.0 [-13.8;-0.3] | 0.04 | -7.8 [-15.2;-0.5] | 0.04 | -0.8 [-2.9;1.3] | 0.45 |
| **Employment status (ref: employed)** | | |  |  |  |  |  |  |  |  |
| Not employed  (student, house maker) | -2.2 [-27.9;23.4] | 0.86 | -13.3 [-34.2;7.6] | 0.21 | -8.2 [-16.3;-0.1] | 0.05 | -8.7 [-17.5;0.2] | 0.05 | -0.5 [-3.0;2.0] | 0.70 |
| Retired, not able to work | 15.4 [-27.7;58.5] | 0.48 | -1.2 [-36.3;33.9] | 0.95 | -9.7 [-23.3;3.9] | 0.16 | -12.1 [-26.9;2.8] | 0.11 | -2.4 [-6.6;1.8] | 0.26 |
| **Monthly net household income (ref: sample average)** | | | |  |  |  |  |  |  |  |
| Above sample average | 1.7 [-24.9;28.3] | 0.90 | 1.2 [-20.4;22.9] | 0.91 | -2.4 [-10.8;6.0] | 0.57 | -2.5 [-11.7;6.6] | 0.59 | -0.1 [-2.7;2.5] | 0.94 |
| Below sample average | 0.6 [-25.3;26.6] | 0.96 | 5.7 [-15.4;26.8] | 0.60 | -7.0 [-15.2;1.2] | 0.09 | -6.5 [-15.4;2.4] | 0.15 | 0.5 [-2.0;3.0] | 0.70 |
| missing | -25.7 [-65.8;14.5] | 0.21 | 14.1 [-18.6;46.7] | 0.40 | 3.5 [-9.1;16.2] | 0.59 | 3.1 [-10.8;16.9] | 0.66 | -0.5 [-4.3;3.4] | 0.82 |
| **BMI (ref: underweight/normal weight)** | | | |  |  |  |  |  |  |  |
| Overweight | 23.6 [2.3;44.9] | 0.03 | -24.6 [-42.0;-7.3] | 0.01 | -3.2 [-9.9;3.5] | 0.35 | -4.7 [-12.0;2.7] | 0.21 | -1.5 [-3.5;0.6] | 0.16 |
| Obesity | 5.9 [-20.0;31.8] | 0.65 | -10.3 [-31.4;10.8] | 0.34 | -3.3 [-11.5;4.8] | 0.43 | -5.5 [-14.4;3.5] | 0.23 | -2.1 [-4.7;0.4] | 0.09 |
| **Smoking behaviour (ref: never smoker)** | | | |  |  |  |  |  |  |  |
| Regular smoker | -31.6 [-61.3;-1.9] | 0.04 | 9.2 [-15.0;33.4] | 0.46 | 3.8 [-5.6;13.2] | 0.43 | 2.7 [-7.5;13.0] | 0.60 | -1.1 [-3.9;1.8] | 0.47 |
| Ex-smoker | -20.9 [-56.1;14.2] | 0.24 | 14.8 [-13.9;43.4] | 0.31 | 2.1 [-9.0;13.2] | 0.71 | 7.3 [-4.8;19.4] | 0.24 | 5.2 [1.8;8.6] | <0.01 |
| **Clinical condition (ref: no chronic disease)** | | |  |  |  |  |  |  |  |  |
| at least 1 chronic disease* | -4.6 [-24.1;14.9] | 0.64 | 2.3 [-13.6;18.2] | 0.77 | 2.4 [-3.7;8.6] | 0.44 | 3.2 [-3.5;9.9] | 0.35 | 0.8 [-1.1;2.7] | 0.43 |

*chronic diseases include: Metabolic diseases, (hypercholesterolemia, diabetes), cardiovascular diseases (Hypertension, Angina pectoris, Heart attack, Stroke, Heart failure, arrhythmia), cancer, lung diseases (asthma, COPD)

**Supplementary Table 2.** Adjusted associations of sociodemographic and health-related factors with inactivity, light physical acitivity (LPA), moderate physical activity (MPA), moderate-to-vigorous physical activity (MVPA), and vigorous physical activity (VPA) among the **Berlin** sample.

| **Variables** | **Inactivity** | | **LPA** | | **MPA** | | **MVPA** | | **VPA** | |
| --- | --- | --- | --- | --- | --- | --- | --- | --- | --- | --- |
|  | minutes per day | | minutes per day | | minutes per day | | minutes per day | | minutes per day | |
|  | Mean difference [95% CI] | p-value | Mean difference [95% CI] | p-value | Mean difference [95% CI] | p-value | Mean difference [95% CI] | p-value | Mean difference [95% CI] | p-value |
| **Sex (ref: female)** | |  |  |  |  |  |  |  |  |  |
| Male | -4.0 [-12.5;4.4] | 0.35 | -9.2 [-16.1;-2.4] | 0.01 | 0.9 [-1.2;3.0] | 0.39 | 1.8 [-0.4;4.0] | 0.11 | 0.9 [0.4;1.4] | <0.01 |
| **Age groups (ref: 20-34 years)** | |  |  |  |  |  |  |  |  |  |
| 35-49 years | -1.1 [-14.2;12.0] | 0.87 | 20.9 [10.2;31.6] | <0.01 | -1.1 [-4.4;2.1] | 0.50 | -0.3 [-3.7;3.1] | 0.85 | 0.8 [0.0;1.5] | 0.04 |
| 50-64 years | 3.2 [-10.8;17.3] | 0.65 | 17.9 [6.5;29.3] | <0.01 | -5.5 [-9.0;-2.1] | <0.01 | -5.7 [-9.3;-2.1] | <0.01 | -0.2 [-1.0;0.6] | 0.70 |
| >64 years | 8.8 [-11.3;29.0] | 0.39 | 17.6 [1.2;34.0] | 0.04 | -11.2 [-16.2;-6.2] | <0.01 | -12.8 [-18.0;-7.6] | <0.01 | -1.6 [-2.7;-0.4] | 0.01 |
| **Education level (ISCED 97) (ref: high)** | | |  |  |  |  |  |  |  |  |
| Middle | -13.4 [-23.4;-3.3] | 0.01 | 12.2 [4.1;20.4] | <0.01 | -1.0 [-3.4;1.5] | 0.45 | -1.4 [-4.0;1.2] | 0.29 | -0.4 [-1.0;0.1] | 0.14 |
| Low | -1.6 [-33.1;29.9] | 0.92 | 9.3 [-16.3;34.9] | 0.48 | -7.4 [-15.2;0.4] | 0.06 | -6.3 [-14.5;1.9] | 0.13 | 1.1 [-0.7;2.9] | 0.21 |
| missing | 0.0 [-13.1;13.1] | 1.00 | 1.6 [-9.0;12.3] | 0.76 | -1.6 [-4.9;1.6] | 0.33 | -1.6 [-5.0;1.8] | 0.36 | 0.0 [-0.7;0.8] | 0.96 |
| **Marital status (ref: married)** | |  |  |  |  |  |  |  |  |  |
| Single | 8.0 [-2.7;18.6] | 0.14 | -25.4 [-34.1;-16.8] | <0.01 | 2.0 [-0.7;4.6] | 0.15 | 2.0 [-0.7;4.8] | 0.15 | 0.1 [-0.6;0.7] | 0.86 |
| Divorced/widdowed | 3.4 [-9.2;16.0] | 0.60 | -13.8 [-24.0;-3.5] | 0.01 | 2.2 [-0.9;5.3] | 0.16 | 2.4 [-0.9;5.6] | 0.15 | 0.2 [-0.6;0.9] | 0.68 |
| **Migration background (ref: no)** | |  |  |  |  |  |  |  |  |  |
| Yes | -6.4 [-17.7;4.9] | 0.27 | 0.4 [-8.8;9.5] | 0.94 | -0.7 [-3.5;2.1] | 0.61 | -0.9 [-3.8;2.0] | 0.55 | -0.2 [-0.8;0.5] | 0.63 |
| **Employment status (ref: employed)** | | |  |  |  |  |  |  |  |  |
| Not employed  (student, house maker) | -6.4 [-26.1;13.3] | 0.52 | -22.7 [-38.7;-6.7] | 0.01 | -8.9 [-13.8;-4.0] | <0.01 | -9.3 [-14.5;-4.2] | <0.01 | -0.5 [-1.6;0.7] | 0.43 |
| Retired, not able to work | -12.2 [-25.5;1.0] | 0.07 | -8.7 [-19.6;2.1] | 0.11 | -8.0 [-11.3;-4.7] | <0.01 | -7.4 [-10.9;-4.0] | <0.01 | 0.6 [-0.2;1.3] | 0.14 |
| **Monthly net household income (ref: sample average)** | | | |  |  |  |  |  |  |  |
| Above sample average | 10.1 [-1.2;21.4] | 0.08 | -11.3 [-20.5;-2.1] | 0.02 | -2.6 [-5.4;0.2] | 0.07 | -2.8 [-5.8;0.1] | 0.06 | -0.2 [-0.9;0.4] | 0.53 |
| Below sample average | -3.3 [-14.6;8.1] | 0.57 | -8.3 [-17.6;0.9] | 0.08 | -1.3 [-4.1;1.5] | 0.36 | -2.1 [-5.1;0.8] | 0.16 | -0.8 [-1.4;-0.1] | 0.02 |
| **BMI (ref: underweight/normal weight)** | | | |  |  |  |  |  |  |  |
| Overweight | 5.1 [-4.4;14.5] | 0.30 | -3.6 [-11.3;4.1] | 0.36 | -1.9 [-4.3;0.4] | 0.11 | -3.5 [-5.9;-1.0] | 0.01 | -1.5 [-2.1;-1.0] | <0.01 |
| Obesity | 23.1 [10.4;35.8] | <0.01 | -17.4 [-27.7;-7.0] | <0.01 | -8.9 [-12.1;-5.8] | <0.01 | -11.5 [-14.8;-8.2] | <0.01 | -2.6 [-3.3;-1.9] | <0.01 |
| **Smoking behaviour (ref: never smoker)** | | |  |  |  |  |  |  |  |  |
| Regular smoker | -10.1 [-20.9;0.7] | 0.07 | -3.2 [-12.0;5.6] | 0.47 | -1.5 [-4.2;1.1] | 0.26 | -2.8 [-5.6;0.0] | 0.05 | -1.2 [-1.9;-0.6] | <0.01 |
| Ex-smoker | 2.6 [-6.9;12.2] | 0.59 | 2.5 [-5.3;10.3] | 0.53 | 1.1 [-1.3;3.4] | 0.38 | 1.0 [-1.5;3.5] | 0.44 | -0.1 [-0.6;0.5] | 0.80 |
| **Clinical condition (ref: no chronic disease)** | | |  |  |  |  |  |  |  |  |
| at least one chronic disease* | 2.3 [-6.9;11.6] | 0.62 | -1.0 [-8.5;6.6] | 0.80 | -1.4 [-3.7;0.9] | 0.22 | -1.8 [-4.2;0.6] | 0.13 | -0.4 [-0.9;0.1] | 0.12 |

*chronic diseases include: Metabolic diseases, (hypercholesterolemia, diabetes), cardiovascular diseases (Hypertension, Angina pectoris, Heart attack, Stroke, Heart failure, arrhythmia), cancer, lung diseases (asthma, COPD)

**Supplementary Table 3.** Accelerometer-measured physical activity (PA)

| **Variables** | **Singapore** | | | **Berlin** | | |
| --- | --- | --- | --- | --- | --- | --- |
|  | Total  (n=1,195) | Women  (n=683) | Men (n=512) | Total  (n=2,060) | Women (n=1,015) | Men (n=1,045) |
|  | mean ± SD | | | | | |
| **Types of activities in minutes per day** | | |  |  |  |  |
| Moderate PA | 64.8 ± 51.1 | 62.2 ± 55.0 | 68.2 ± 45.1 | 55.9 ± 24.6 | 56.1 ± 23.9 | 55.7 ± 25.2 |
| Vigorous PA | 1.9 ± 15.6 | 0.96 ± 2.3 | 3.03 ± 23.6 | 2.2 ± 5.6 | 1.9 ± 5.3 | 2.4 ± 5.8 |
| 10-min-bouted MVPA* | 20.8 ± 50.6 | 20.6 ± 52.1 | 21.2 ± 48.5 | 16.2 ± 17.6 | 16.3 ± 18.3 | 16.12 ± 17.0 |

*Moderate-to-vigorous physical activity accumulated in continuous bouts of at least 10 minutes.

**Supplementary Table 4.** Weekday distribution of physical activity and inactivity for Singapore and Berlin

|  | Monday | Tuesday | Wednesday | Thursday | Friday | Saturday | Sunday |
| --- | --- | --- | --- | --- | --- | --- | --- |
|  | Time spent in activity or inactivity in % per day | | | | | | |
| Singapore |  |  |  |  |  |  |  |
| N | 1062 | 1086 | 1070 | 1084 | 1070 | 1060 | 1060 |
| MVPA | 8.0 ± 6.6 | 8.3 ± 8.0 | 8.3 ± 7.6 | 8.4 ± 7.8 | 8.5 ± 7.8 | 8.4 ± 8.6 | 8.0 ± 8.0 |
| LPA | 31.5 ± 18.6 | 31.8 ± 19.3 | 31.1 ± 18.4 | 31.4 ± 19.0 | 32.8 ± 19.4 | 32.7 ± 17.9 | 32.4 ± 17.5 |
| inactivity | 60.4 ± 20.6 | 59.9 ± 21.8 | 60.5 ± 20.7 | 60.2 ± 21.3 | 58.8 ± 21.4 | 58.9 ± 20.3 | 59.5 ± 20.1 |
| Berlin |  |  |  |  |  |  |  |
| N | 1596 | 1595 | 1566 | 1615 | 1680 | 1956 | 1956 |
| MVPA | 6.9 ± 4.0 | 7.1 ± 4.2 | 6.9 ± 4.1 | 7.0 ± 4.1 | 6.9 ± 4.0 | 6.8 ± 4.6 | 6.3 ± 4.9 |
| LPA | 23.2 ± 11.5 | 23.1 ± 11.1 | 22.5 ± 10.4 | 22.8 ± 10.6 | 24.3 ± 11.3 | 26.0 ± 11.3 | 23.8 ± 11.7 |
| inactivity | 69.9 ± 12.8 | 69.8 ± 12.4 | 70.6 ± 11.8 | 70.3 ± 12.2 | 68.8 ± 12.8 | 67.2 ± 12.9 | 69.9 ± 13.5 |


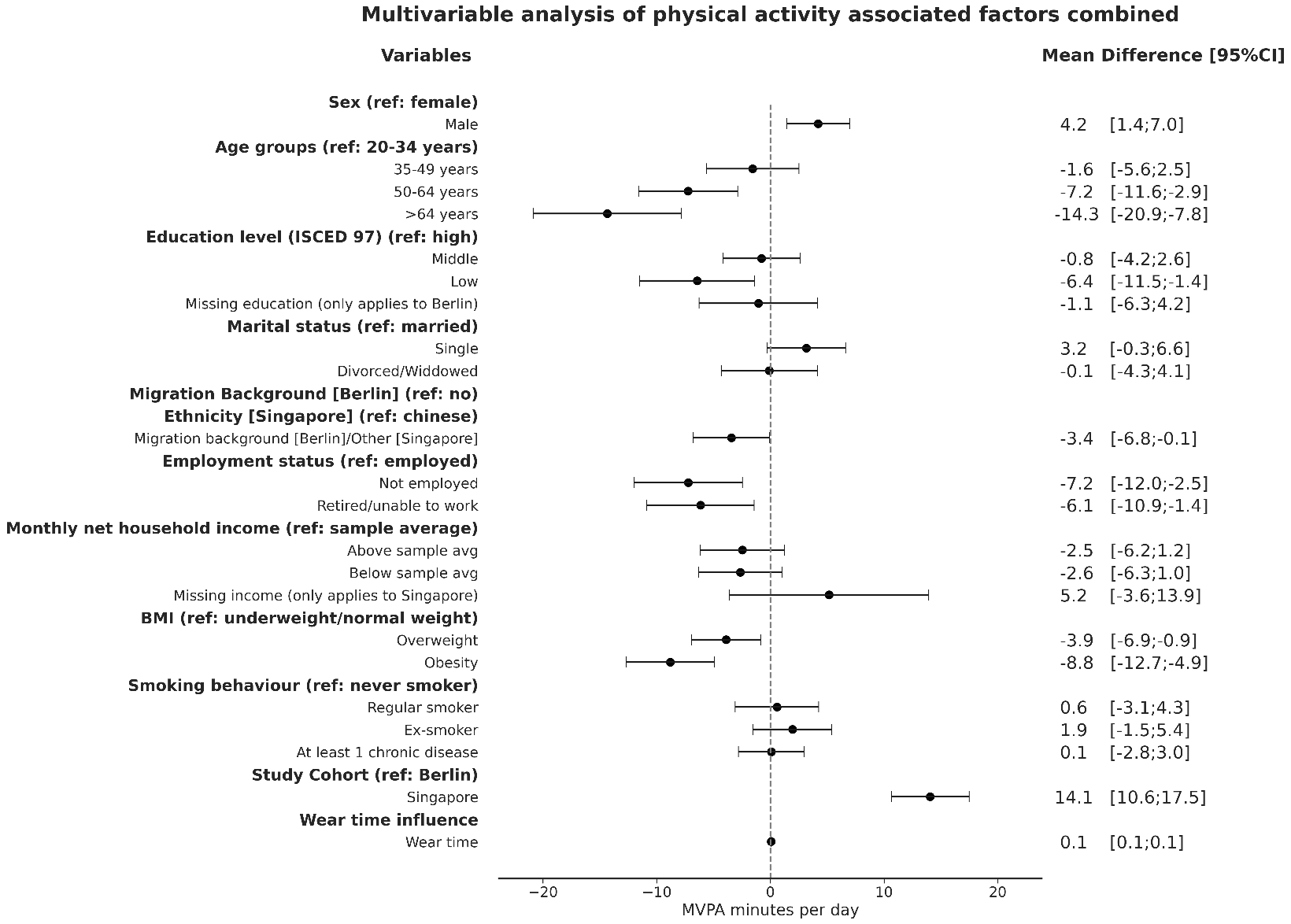


**Supplementary Figure 2.** Multivariable regression analysis (mutually adjusted for all covariates) for the complete study sample (Singapore and Berlin). **Outcome: Moderate-to-vigorous physical activity (MVPA)**-minutes per day. The coefficient represents mean differences in MVPA-minutes with 95% confidence intervals.

**
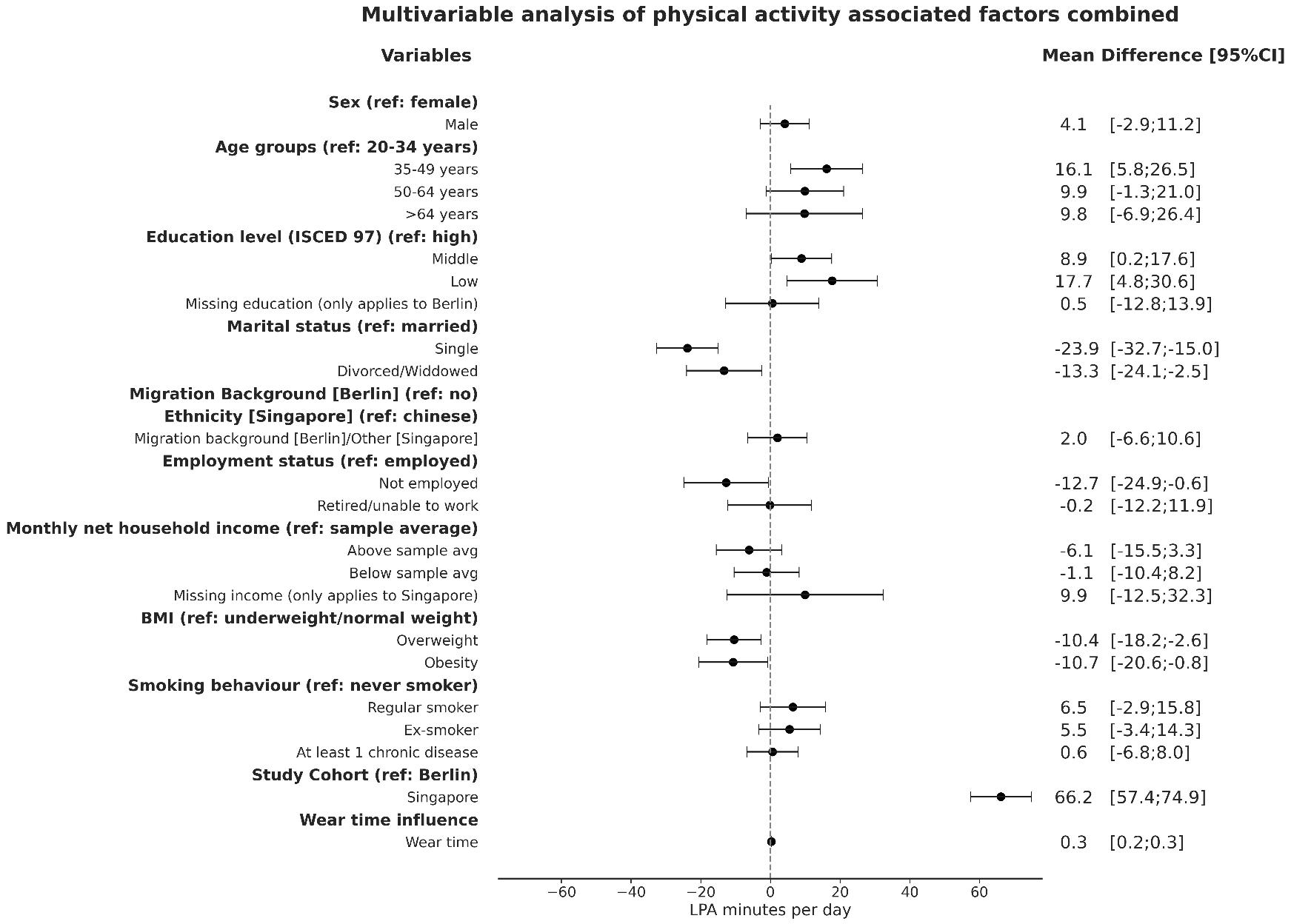
**

**Supplementary Figure 3.** Multivariable regression analysis (mutually adjusted for all covariates) for the complete study sample (Singapore and Berlin). **Outcome: Light physical activity (LPA)**-minutes per day. The coefficient represents mean differences in LPA minutes with 95% confidence intervals.

**
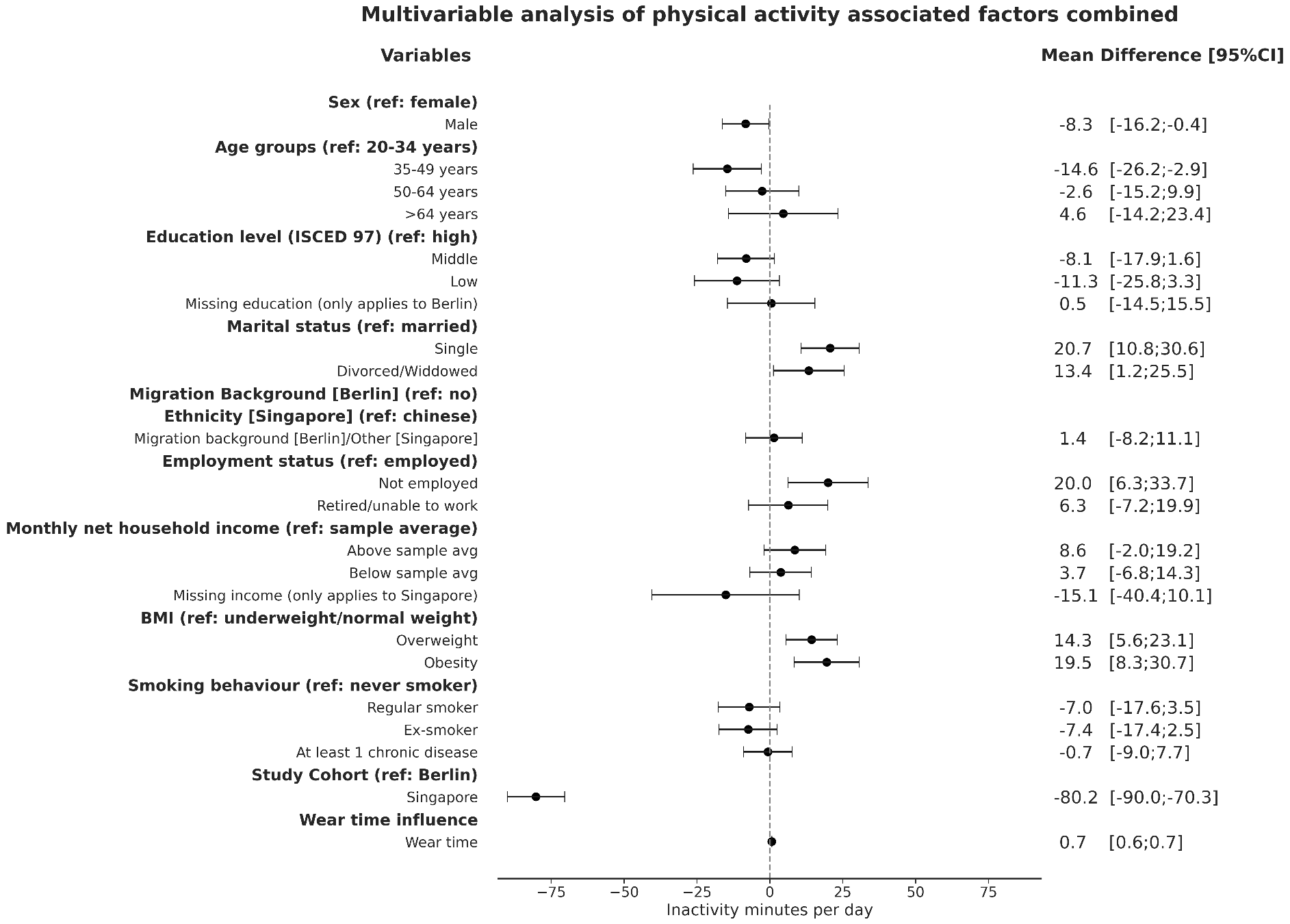
**

**Supplementary Figure 4.** Multivariable regression analysis (mutually adjusted for all covariates) for the complete study sample (Singapore and Berlin). **Outcome: Inactivity** minutes per day. The coefficient represents mean differences in inactivity minutes with 95% confidence intervals.
